# Tie-2 regulates the stemness and metastatic properties of prostate cancer cells

**DOI:** 10.18632/oncotarget.3950

**Published:** 2015-04-29

**Authors:** Kai-Dun Tang, Boris M. Holzapfel, Ji Liu, Terence Kin-Wah Lee, Stephanie Ma, Lidija Jovanovic, Jiyuan An, Pamela J. Russell, Judith A. Clements, Dietmar W. Hutmacher, Ming-Tat Ling

**Affiliations:** ^1^ Australian Prostate Cancer Research Centre-Queensland and Institute of Health and Biomedical Innovation, Queensland University of Technology and Translational Research Institute, Woolloongabba, Qld, Australia; ^2^ Department of Pathology, Faculty of Medicine, The University of Hong Kong, Hong Kong, SAR, China; ^3^ Department of Anatomy, Faculty of Medicine, The University of Hong Kong, Hong Kong, SAR, China

**Keywords:** Tie-2, prostate cancer, metastasis, cancer stem cells

## Abstract

Ample evidence supports that prostate tumor metastasis originates from a rare population of cancer cells, known as cancer stem cells (CSCs). Unfortunately, little is known about the identity of these cells, making it difficult to target the metastatic prostate tumor. Here, for the first time, we report the identification of a rare population of prostate cancer cells that express the Tie-2 protein. We found that this Tie-2^High^ population exists mainly in prostate cancer cell lines that are capable of metastasizing to the bone. These cells not only express a higher level of CSC markers but also demonstrate enhanced resistance to the chemotherapeutic drug Cabazitaxel. In addition, knockdown of the expression of the Tie-2 ligand angiopoietin (Ang-1) led to suppression of CSC markers, suggesting that the Ang-1/Tie-2 signaling pathway functions as an autocrine loop for the maintenance of prostate CSCs. More importantly, we found that Tie-2^High^ prostate cancer cells are more adhesive than the Tie-2^Low^ population to both osteoblasts and endothelial cells. Moreover, only the Tie-2^High^, but not the Tie-2^Low^ cells developed tumor metastasis *in vivo* when injected at a low number. Taken together, our data suggest that Tie-2 may play an important role during the development of prostate tumor metastasis.

## INTRODUCTION

Prostate cancer is one of the most common solid tumors in men and is the second leading cause of morbidity and mortality in men worldwide [1, 2]. Patients with advanced prostate cancer are normally treated with hormone ablation therapy. This therapy is effective initially, as prostate cancer cells require androgen to grow and survive; however, the cancer cells eventually become androgen-independent and develop metastatic, castration-resistant tumors [3]. At this stage, chemotherapy and radiotherapy have only minor benefits. As a result, advanced prostate cancer remains incurable with current treatment strategies [4].

Recent studies suggested that prostate tumor-initiating cells (TICs)/cancer stem cells (CSCs) may play key roles in the initiation, progression and treatment failure of the disease [5–7]; however, their exact identity remains unclear. These cells share many similarities with normal stem cells, including the ability to differentiate into cells of different lineages and the dependence on a stem cell niche for the maintenance of their stemness. Interestingly, according to previous studies, CSCs are capable of creating their own CSC niche by recruiting mesenchymal stem cells derived from bone marrow [8]. CSCs can also ‘hijack’ the established normal stem cell niches [9]; as has been demonstrated in both human and mouse models of prostate and breast cancers. According to Shiozawa et al. [10], prostate CSCs are capable of competing with hematopoietic stem cells (HSCs) for the bone marrow niche. Moreover, prostate cancer cells that occupy the niche were found to express the CXCR4 receptor, which is responsible for mediating the interaction between HSCs and the niche. By blocking the CXCL12/CXCR4 pathway, prostate cancer bone metastasis was significantly suppressed, supporting the hypothesis that prostate tumors metastasize to the bone by adopting this bone-homing signaling pathway. However, although disseminated prostate cancer cells can be detected in the bone marrow of prostate cancer patients, bone metastasis may take years to develop. This may reflect the time required for the disseminated cancer cells to establish a suitable tumor microenvironment before the cells can grow exponentially. Therefore, a better understanding of the bone marrow stem cell niche and its role in supporting bone metastasis may aid the development of effective treatments.

Tie-2 is a membrane receptor commonly expressed by HSCs, osteoblasts and endothelial cells. It binds to and is activated by angiopoietin-1 (Ang-1), a cytokine actively secreted by osteoblasts within the bone marrow niche [11–13]. Similar to the KITLG/c-Kit, CXCL12/CXCR4 and FGF1/FGFR chemokine axes, the Ang-1/Tie-2 signalling pathway also plays a key role in regulating the homing and stemness of HSCs [14–18]. According to Arai et al. [15], Tie-2 receptors are expressed by the quiescent HSCs that adhere to osteoblasts within the inner surface of the bone. The same group further demonstrated that osteoblasts secrete a large amount of Ang-1, which promotes the adhesion of HSCs to osteoblasts while maintaining the cells in a quiescent state. Recently, the Ang-1/Tie-2 signalling pathway was also found to promote muscle satellite cell self-renewal [19]. Apart from regulating normal stem cells, Tie-2 was also found to play a role in cancer progression. As reported by Lee et al. [20], Tie-2 was found to be expressed by neoplastic glial cells, and its expression level was significantly associated with disease progression. Tie-2 activation was also found to correlate with chemoresistance [21]. Further research by the same group demonstrated that Tie-2 promoted glioma cell invasion and modulated the interaction of glioma tumor stem cells with endothelial cells [22].

In this study, we show that Tie-2 is expressed by a rare population of prostate cancer cells that co-express several CSC markers. Compared to the Tie-2^Low^ population, Tie-2^High^ prostate cancer cells demonstrate not only enhanced chemoresistance but also an increased ability to adhere to stromal cells such as endothelial cells and osteoblasts. Intra-cardiac injection of the cells into immune-incompetent mice confirmed that Tie-2^High^ cells, but not Tie-2^Low^ cells, actively developed into metastatic tumors *in vivo*. Our data, therefore, have demonstrated a novel role for Tie-2 in the development of prostate tumor metastasis.

## RESULTS

### Identification of a rare population of Tie-2^High^ prostate cancer cells

**Figure 1:**
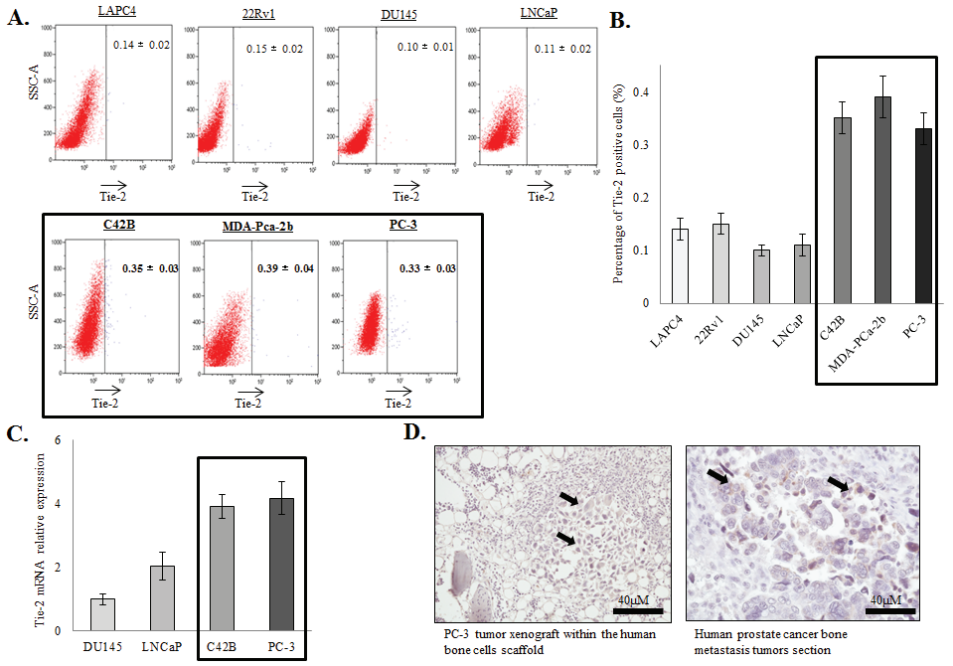
Expression of Tie-2 in prostate cancer cells **A.**&**B.** Flow cytometry analysis of Tie-2 expression in a panel of prostate cancer cell lines (LAPC4, 22Rv1, DU145, LNCaP, C42B, MDA-PCa-2b and PC-3). The results are presented as the mean ± SD from triplicate experiments. **C.** Tie-2 mRNA was analyzed in these different prostate cancer cell lines using qRT-PCR. Note that Tie-2 protein and mRNA expression were both increased in bone metastatic prostate cancer cell lines (highlighted). Results quantified represented as fold change normalized to DU145. **D.** Immunohistochemical staining was performed on humanized bone scaffold containing the PC-3 metastastic tumor (5 cases) (left panel) and human bone section containing the metastatic prostate tumor (1 case) (right panel). All the sections were stained with antibody against Tie-2. Note that, all the sections were positive for the Tie-2 proteins and the arrows showed the positive staining within the tumor cells (40X magnifications).

Although Ang-1/Tie-2 is one of the key signalling pathways involved in HSC maintenance, it is currently unknown whether it plays any role in prostate cancer progression. To address this question, we first examined whether prostate cancer cells express Tie-2. Using a PE-conjugated anti Tie-2 antibody, the expression level of the Tie-2 receptor in a panel of prostate cancer cell lines with different metastatic potential (LAPC4, 22Rv1, DU145, LNCaP, C42B, MDA-PCa-2b and PC-3) was determined by flow cytometry. As shown in Figure 1A and 1B, Tie-2 positive cells exist at a very low level in soft tissue metastatic prostate cancer cell lines (between 0.1 - 0.15%). However, the Tie-2 positive population was found higher in C42B (a bone metastatic cell line derived from LNCaP) (0.35%), and two bone metastatic prostate cancer cell lines (0.33% and 0.39% in PC-3 and MDA-PCa-2b respectively). Consistently, qRT-PCR analysis revealed that Tie-2 mRNA was expressed at a higher level in the highly metastatic prostate cancer cell lines (Figure 1C). More importantly, Tie-2 was also found to be expressed not only in PC-3 tumors metastasized into a humanized bone scaffold (left panel) established in our previous study [23], but also in bone metastatic tumor of human prostate cancer patient (right panel), further suggesting that Tie-2 may play roles in the development of prostate tumor bone metastasis (Figure 1D).

### Co-expression of stem cell markers with Tie-2 in prostate cancer cells

**Figure 2:**
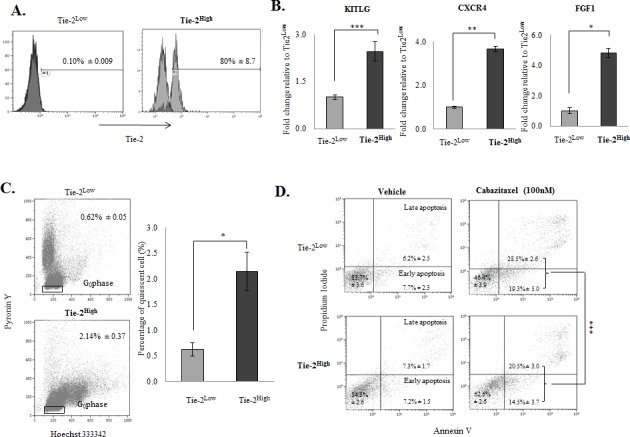
The Tie-2^High^ population possessed stem cell characteristics **A.** Analysis of the population sorted by flow cytometry confirmed the successful enrichment of Tie-2^High^ cells. **B.** Validation of the selected candidate genes (i.e., KITLG, CXCR4, and FGF1) with qRT-PCR. Results were normalized with internal control and are presented as fold change relative to Tie-2^Low^ population. **C.** Flow cytometry analysis revealed that quiescent cells were increased by more than 3-fold in the Tie-2^High^ population when compared to the Tie-2^Low^population. **D.** Flow cytometry analysis of apoptotic cells by Annexin V staining in Tie-2^Low^ and Tie-2^High^ cells that were treated with 100nM Cabazitaxel for 72hrs (*p* value = 0.0018 for apoptosis). Note that a high percentage of apoptotic cells were detected in the Tie-2^Low^ population when compared to the Tie-2^High^ prostate cancer cells. (*p* values: * < 0.05, ** < 0.005, *** < 0.0005).

To further characterize the Tie-2 positive prostate cancer cells, we first performed FACS to enrich the Tie-2-positive population from PC-3 cells. Analysis of the population sorted by flow cytometry confirmed the successful enrichment of Tie-2^High^ cells (Figure 2A) by cell sorting. cDNA microarray analysis was then performed to examine the gene expression profile of Tie-2^High^ cells. As shown in in Suppl Table 2, several stem cell factors/markers commonly found in HSCs (e.g. FGF1, KITLG and CXCR4) were found to be upregulated in the Tie-2^High^ population, which was further confirmed by qRT-PCR analysis (Figure 2B), demonstrating that Tie-2 expression is associated with upregulation of HSC markers.

### Tie-2 regulates the quiescence of prostate cancer cells

One of the key roles of Tie-2 is to regulate the quiescence state of HSCs. To determine if expression of Tie-2 is associated with cellular quiescent, HO/PY staining was performed to quantitate quiescent population in both Tie-2^High^ and Tie-2^Low^ prostate cancer cells. As expected, the population of quiescent cells was increased more than 3-fold in the Tie-2^High^ population when compared to the Tie-2^Low^ population (Figure 2C), suggesting that Tie-2 expression plays an important role in maintaining the quiescent state of prostate cancer cells. Cellular quiescence has been shown to contribute to the chemodrug resistance of CSCs. We therefore examined the sensitivity Tie-2^High^ prostate cancer cells to Cabazitaxel, a chemotherapeutic drug commonly used for the treatment of prostate cancer. As shown in Figure 2D, treatment of the Tie-2^Low^ population with Cabazitaxel led to induction of apoptosis of 48% cells, as evidenced by Annexin V staining. However, the apoptotic population was significantly lower in Tie-2^High^ cells under the same conditions (<35%), clearly demonstrating that Tie-2 expression is associated with Cabazitaxel resistance.

### Ang-1 activates the Tie-2 downstream signalling pathway in prostate cancer cells

**Figure 3:**
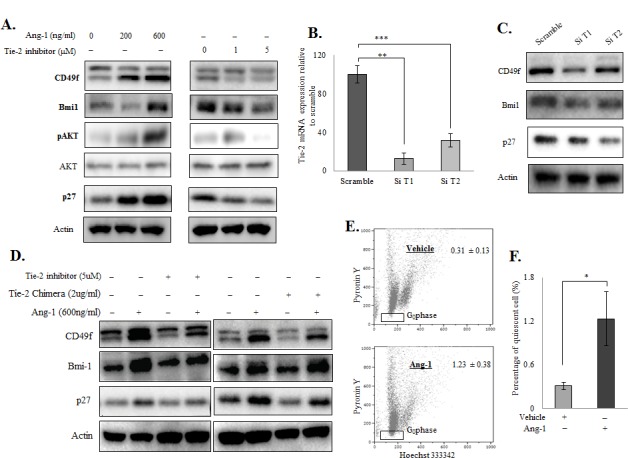
Ang-1 upregulated prostate CSC and quiescent markers in prostate cancer cell lines Western blotting **A.** of prostate CSC markers (CD49f and Bmi-1) and a quiescence marker (p27) after Ang-1 treatment in PC-3 cells (left panel). Ang-1 was found to upregulate both stem cell and quiescence markers in a dose-dependent manner. The Tie-2 inhibitor, on the other hand, suppressed both types of markers in a dose-dependent manner in PC-3 cells (right panel). **B.** Transfection of Tie-2 siRNAs led to downregulation Tie-2 mRNA levels in PC-3 cells. Results were normalized with internal control and are presented as fold change relative to scramble. **C.** Effect of Tie-2 knockdown on CSC and quiescence marker expression in PC-3 cells. **D.** The experiment was repeated in the presence of Tie-2 inhibitor (left panel) or a Tie-2 neutralizing antibody (right panel), which showed that both the Tie-2 inhibitor and Tie-2 neutralizing antibody abolished the effect of Ang-1 on PC-3 cells. **E.** Quantitation of the quiescent population in PC-3 cells with or without Ang-1 treatment. Note that Ang-1 treatment led to a 4-fold induction of the quiescent population in PC-3 cells when compared to the vehicle control. Each experiment was repeated at least three times, and the results are presented as the mean ± SD. (*p* values: * < 0.05, ** < 0.005, *** < 0.0005).

Because the Ang-1/Tie-2 signalling cascade plays a role in the regulation of HSC stemness, we therefore questioned whether Ang-1 also regulates the stemness of prostate cancer cells. We first treated PC-3 cells with increasing doses of recombinant Ang-1 (0, 200 and 600 ng/ml) for 72 hours under serum-free conditions. The expression of a series of stem cell factors/markers known to be induced by the Ang-1/Tie-2 signalling in HSCs was then examined by Western blotting. As shown in Figure 3A (left panel), Ang-1 induced a dose-dependent increase in AKT phosphorylation, a direct downstream target of the Ang-1/Tie-2 signalling pathway, confirming that Tie-2 activates prostate cancer cells. More importantly, Ang-1 treatment was found to induce the expression of prostate CSC (CD49f and Bmi-1) and quiescence (p27) markers in PC-3 cells in a dose-dependent manner. When the cells were treated with a Tie-2 kinase inhibitor (0, 1 and 5 μM), a cell permeabile pyridinylimidazole found to block the kinase activity of Tie-2 [24], all the markers tested were found to be downregulated, suggesting that activation of Tie-2 is required for maintaining the levels of these markers (Figure 3A, right panel). To confirm our findings, cells were transfected with two different siRNAs that target different regions of the Tie-2 mRNA, which resulted in a significant decrease (>50%) in the Tie-2 mRNA level (Figure 3B). As shown in Figure 3C, knockdown of Tie-2 led to concomitant decrease in the level of CD49f, Bmi-1 as well as p27. More interestingly, the effect of recombinant Ang-1 was significantly suppressed when the cells were pre-treated with the Tie-2 inhibitor (Figure 3D, left panel) or Tie-2 Fc Chimera (Tie-2 neutralizing peptide) (Figure 3D, right panel). Furthermore, ectopic expression of Tie-2 in DU145 cells, which lack endogenous Tie-2 expression (Suppl Figure 1A) and fail to respond to Ang-1 (data not shown), was found to successfully restore the response of the cells to Ang-1 treatment (Suppl Figure 1B), further supporting the hypothesis that activation of Tie-2 by Ang-1 is crucial for maintaining the expression of stem cell markers in prostate cancer cells.

Since Ang-1 regulates the stemness of HSCs through induction of cellular quiescence, we therefore examined if Ang-1 treatment also induces quiescent of the prostate cancer cells. PC-3 cells were first treated with Ang-1 before being stained with HO/PY for quantitation of quiescent population. As shown in Figure 3E&3F, when cells were treated with recombinant Ang-1, a 4-fold increase in the quiescent population was observed. Consistently, expression of p27, a protein closely associated with cell cycle arrest and cellular quiescence, was also found to be induced by Ang-1 treatment in PC-3 cells (Figure 3A). Meanwhile, inactivation of Tie-2 using siRNA (Figure 3C), a Tie-2-specific inhibitor or a Tie-2 Fc chimera (Figure 3D) also led to suppression of p27, even in the presence of recombinant Ang-1, further suggesting that Ang-1 induces quiescence of prostate cancer cells through the activation of Tie-2.

### Ang-1 functions as a novel autocrine factor in prostate cancer cells

**Figure 4:**
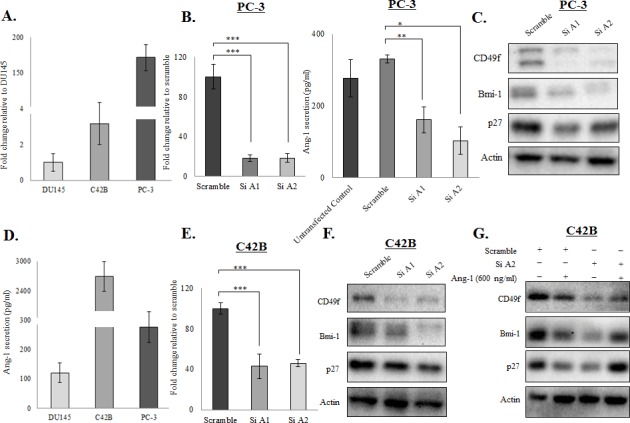
Ang-1 functioned as an autocrine factor in prostate cancer cells **A.** Ang-1 mRNA expression was determined in different prostate cancer cell lines (DU145, C42B and PC-3) using qRT-PCR analysis. Results were normalized with internal control and are presented as fold change relative to DU145. **B.** Knockdown of Ang-1 in PC-3 cells by siRNA transfection was confirmed with qRT-PCR and ELISA. Note that Ang-1 expression was suppressed by >80% in PC-3 cells. Results were normalized with internal control and are presented as fold change relative to scramble. **C.** Downregulation of Ang-1 in PC-3 cells by siRNA was associated with suppression of CSC (CD49f and Bmi-1) and quiescence (p27) markers. **D.** Ang-1 secretion (pg/ml) by different prostate cancer cell lines (DU145, C42B and PC-3) was determined with an ELISA. **E.** Knockdown of Ang-1 for >50% was confirmed with RT-PCR in C42B cells. **F.** Ang-1 knockdown suppressed both CSC (CD49f and Bmi-1) and quiescence (p27) markers in C42B cells. **G.** The response of C42B cells to exogenous Ang-1 treatment (600ng/ml) was restored when endogenous Ang-1 was knocked down by siRNA. (*p* values: * < 0.05, ** < 0.005, *** < 0.0005).

Because prostate cancer cells are known to secrete Ang-1, we hypothesized that Ang-1 may indeed function as an autocrine factor in prostate cancer cells. To test this hypothesis, we first confirmed the expression of Ang-1 mRNA in the prostate cancer cell lines with qRT-PCR. Consistent with previous studies, Ang-1 mRNA was detectable in prostate cancer cell lines, and its expression is found higher in metastatic prostate cancer cell lines (Figure 4A). Nevertheless, transfection of PC-3 cells with two different Ang-1 siRNAs led to >80% suppression of the Ang-1 mRNA level, as shown in Figure 4B. More importantly, knockdown of endogenous Ang-1 resulted in suppression of both CSC (CD49f an Bmi-1) and quiescence (p27) markers in PC-3, supporting the hypothesis that Ang-1 produced by cancer cells is crucial for CSC maintenance (Figure 4C). Surprisingly, examination of Ang-1 secretion with ELISA revealed that C42B produced the highest level of Ang-1 among all the prostate cancer cell lines (Figure 4D). Meanwhile, we found that C42B cells failed to respond to treatment with Ang-1 recombinant proteins (data not shown); however, knockdown of endogenous Ang-1 not only suppressed the expression of CD49f an Bmi-1, but also restored the response of the cells to exogenous Ang-1 treatment, as evidenced by the induction of CSC and quiescence markers by recombinant Ang-1 (Figure 4E–4G). These findings further confirm that Ang-1 regulates the stemness of prostate cancer cells by functioning as an autocrine factor.

### Tie-2 facilitates the adhesion of prostate cancer cells to osteoblasts and endothelial cells

**Figure 5:**
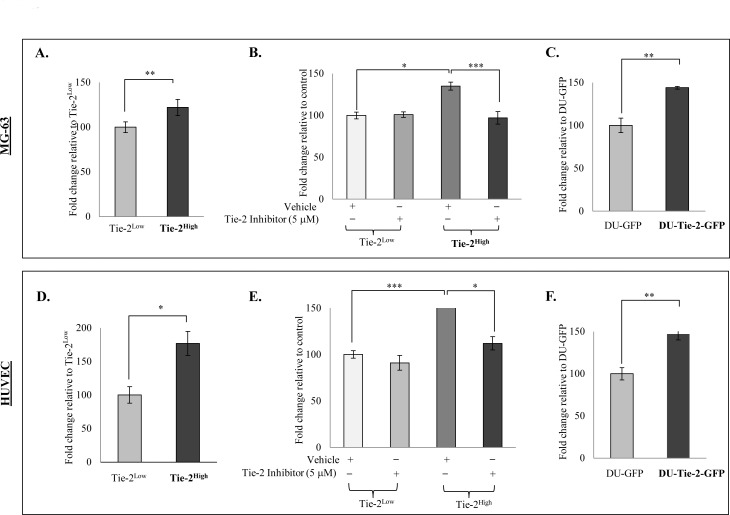
Tie-2 facilitated the adhesion of prostate cancer cells to osteoblasts and endothelial cells A cell adhesion assay was performed with the sorted Tie-2^High^ and Tie-2^Low^ PC-3 cells prestained with Hoechst 33342. Cells that adhered to osteoblasts (MG-63) **A.** or endothelial cells (HUVECs) **D.** were quantified by measuring the fluorescence intensity, and the results are presented as the mean ± SD. Note that Tie-2^High^ PC-3 cells were more adhesive to MG-63 and HUVECs when compared to Tie-2^Low^PC-3 cells. **B**&**E.** Effect of Tie-2 inactivation on the adhesive ability of Tie-2^High^ PC-3 cells. Treatment with a Tie-2 inhibitor (5 μM) prior to the adhesion assay significantly suppressed the adhesion ability of Tie-2^High^ cells, while the same treatment failed to affect the Tie-2^Low^ population. **C**&**F.** Ectopic Tie-2 expression promoted the adhesion of DU145 cells to osteoblast MG-63 cells and HUVECs. Each experiment was repeated at least three times, and the results are presented as the mean ± SD. (*p* values: * < 0.05, ** < 0.005, *** < 0.0005).

One of the functions of Tie-2 in HSC maintenance is to facilitate the adhesion of HSCs to osteoblasts, which is a process that is also required by prostate cancer cells during the development of bone metastasis. This led us to speculate that Tie-2 may also regulate the adhesion of prostate cancer cells to osteoblasts. The ability of the Tie-2^High^ population to adhere to osteoblasts was then determined with a cell adhesion assay using the osteosarcoma cell line (MG-63). Examination of the cells that adhered to a confluent monolayer of MG-63 cells revealed a significant increase in the adhesion ability of the Tie-2^High^ population to osteoblasts when compared to the Tie-2^Low^ cells (Figure 5A). This adhesion ability was completely abolished in the presence of the Tie-2 inhibitor, although the same treatment failed to affect the adhesion ability of the Tie-2^Low^population (Figure 5B). A similar result was observed in another osteosarcoma cell line (SaOS-2) (Suppl Figure 2A & 2B). Strikingly, we found that the Tie-2^High^ population also showed an increased ability to adhere to endothelial cells (Figure 5D), which again could be abolished by the addition of the Tie-2 inhibitor (Figure 5E). To determine whether this effect of Tie-2 is specific to PC-3 cells, we repeated the cell adhesion assay with DU-Tie-2-GFP. Similar to Tie-2^High^ PC-3 cells, DU-Tie-2-GFP cells also demonstrated increased adhesion to both MG-63 and HUVECs when compared to the control cells (DU-GFP), as shown in Figure 5C & 5F. Meanwhile, the adhesion ability of DU-Tie-2-GFP cells to MG-63 and HUVECs was also able to abolish by the addition of Tie-2 inhibitor as shown in Suppl Figure 2C & 2D. Recently, Ang-1 has been shown to promote the bridging of intercellular Tie-2 [25]. Considering that osteoblasts express high levels of Tie-2, it is possible that the cell adhesive ability of the Tie-2 positive prostate cancer cells is mediated through bridging of the intercellular Tie-2. Nevertheless, these findings provide strong evidence that Tie-2 promotes the adhesion of prostate cancer cells to both endothelial cells and osteoblasts.

### Tie-2 enriched cells are highly metastatic *in vivo*

**Figure 6:**
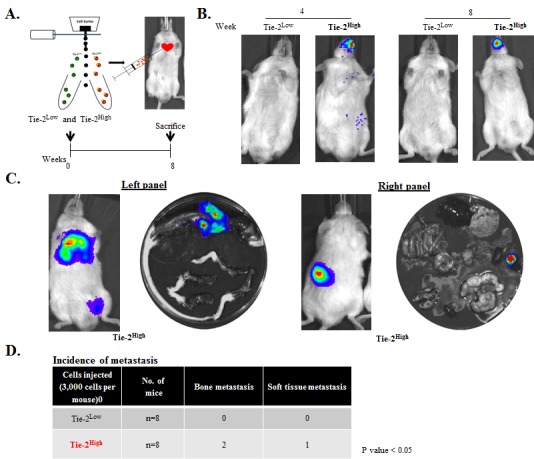
The Tie-2^High^ population was highly metastatic *in vivo* **A.** Tie-2^High^ and Tie-2^Low^ populations sorted out by FACS were injected into mice via intracardiac injection (top). An experimental regimen showing the intracardiac implantation and monitoring of tumor metastasis is shown below. **B.** Representative bioluminescence images of a mouse from each group 4 and 8 weeks after the implantation. **C.** *Ex vivo* imaging of the Tie-2^High^ metastatic tumors. **D.** Summary of the metastatic tumors detected in each mouse. Three out of the eight mice that were injected with Tie-2^High^ cells exhibited metastasis (two in the bone and one in the kidney) (*p* < 0.05). (See also Suppl Figure 3).

Because we found that higher levels of Tie-2 were expressed in metastatic prostate cancer cell lines, and conferred the preferential ability to induce a stem cell-like phenotype and cell adhesion ability of the cancer cells, we hypothesized that Tie-2 may play an important role in prostate tumor metastasis. To test this hypothesis, we examined the ability of Tie-2^High^ prostate cancer cells to form metastatic tumors *in vivo*. Three thousand Tie-2^High^ and Tie-2^Low^ cells isolated from the PC-3-Luc cell line were injected into the mice via intra-cardiac injection as shown in Figure 6A. Subsequent development of metastatic tumors was then determined by bioluminescence (Figure 6B) and *ex vivo* (Figure 6C and Suppl Figure 3) imaging. While mice injected with the Tie-2^Low^ cells failed to develop any tumors, metastatic tumors were found in 3 out of the 8 mice that were injected with the Tie-2^High^ population. Of the 3 mice that developed tumors, two exhibited bone metastasis and one exhibited soft tissue metastasis to the kidney, as shown in Figure 6D. These data strongly support that Tie-2^High^ prostate cancer cells are highly metastatic and have the ability to form metastatic tumors *in vivo*.

## DISCUSSION

In this study, we showed that a rare population of Tie-2-positive cells are present in bone metastatic prostate cancer cell lines (PC-3, C42b and MDA-PCa-2b). These cells not only demonstrated an increased ability to adhere to osteoblasts and endothelial cells but were also found to be more resistant to chemotherapeutic drugs when compared to the Tie-2^Low^ population. More importantly, we found that these cells were capable of developing into metastatic tumors *in vivo*, even when a low number of cells were injected. These cells may thus represent the prostate CSC-like population, which contributes to treatment failure and disease metastasis.

Ample evidence has suggested that CSCs isolated from solid tumors share similar characteristics with HSCs [26–28]. Indeed, several signalling pathways involved in HSC maintenance were also found to be activated in CSCs. For example, CXCL12/CXCR4 was found to be a key regulator of tumor dissemination in cancer cells [29–31], including prostate cancer cells [10, 32] and it also plays an important role in the activation of prostate progenitor cells [33]. Recently, KITLG/c-Kit was found to be involved in CSC maintenance in different cancer types, including prostate cancer [34–37]. Moreover, these studies also showed that the c-Kit receptor was highly expressed in human prostate tumors that metastasized to the bone [38]. It is therefore not surprising that the Ang-1/Tie-2 signaling pathway may also play a key role in regulating the stemness of prostate CSCs, particularly because Tie-2 was found to be expressed by glioma stem cell populations [22]. Indeed, our results suggest that activation of Tie-2 may be required for maintaining both the stemness and quiescent state of prostate cancer cells. The finding that the Tie-2^High^ population was more resistant to Cabazitaxel further supports this notion. Meanwhile, we also observed a significant increase in HSC factors/markers (KITLG, CXCR4 and FGF1) in Tie-2^High^ PC-3 cells when compared to the Tie-2^Low^ population, further suggesting that Tie-2^High^ prostate cancer cells are similar to HSCs.

Prostate cancer cells are known to actively secrete a large amount of Ang-1, which induces tumor angiogenesis by binding to and activating Tie-2 in endothelial cells [39, 40]. Our finding that prostate cancer cells also express functional Tie-2 suggests that Ang-1 may also function through an autocrine loop. Indeed, knockdown of either Ang-1 or Tie-2 was found to downregulate CSC and quiescence markers in PC-3 and C42B cells. Further support was derived from the finding that C42B cells, which express the highest level of Ang-1, failed to respond to exogenous Ang-1 unless endogenous Ang-1 was knocked down by siRNA transfection (data not shown). It is worth noting that autocrine loops such as KITLG/c-Kit and CXCL12/CXCR4 may also exist, although their underlying functions and therapeutic potential remain to be elucidated.

Apart from regulating the stemness of HSC, the Ang-1/Tie-2 pathway is also known to facilitate the adhesion of HSCs to the osteoblastic niche [15]. Similarly, in our *in vitro* adhesion assays, compared to Tie-2^Low^ cells, Tie-2^High^ PC-3 cells were more adhesive to osteosarcoma MG-63 and SaOS-2 cells. This result suggests that Tie-2 receptor may also play a key role in mediating the adhesion of prostate cancer cells to osteoblasts. This possibility was further confirmed by our finding that Tie-2 overexpression promoted the adhesion of DU145 cells to osteoblast cells. Interestingly, similar effects were also observed in endothelial cells, where Tie-2^High^ PC-3 cells showed increased adhesion to endothelial cells, where Tie-2^High^ PC-3 showed increased adhesion to endothelial cells. Because both intravasation and extravasation of tumor cells required their active adhesion to endothelial cells [41, 42], it is conceivable that Tie-2 may play roles in both processes during the development of prostate tumor metastasis and that Tie-2^High^ prostate cancer cells are likely to be more metastatic. This was indeed confirmed by the finding that injection of only 3,000 Tie-2^High^ cells could induce the formation of metastatic tumors *in vivo*. Interestingly, kidney metastasis was found in one of the mice, which may reflect the rich blood supply of the kidney tissue. Indeed, sub-renal capsule grafting has been shown to provide the optimum microenvironment for tumor growth, and was most efficient in terms of uptake rate (>90%) for both benign and malignant prostate tissues [43].

In summary, we have demonstrated for the first time that Tie-2 is expressed by a rare population of prostate cancer cells and plays an important role in regulating the stemness and metastatic ability of the cells (summarized in Suppl Figure 4). Our results highlight the therapeutic potential of targeting Tie-2 with existing inhibitors for the treatment of metastatic prostate cancer, which warrants further investigation.

## MATERIALS AND METHODS

### qRT-PCR analysis

Total RNA was isolated using an RNeasy Mini Kit (Qiagen, Germantown, MD, USA) following the manufacturer's instructions. Two micrograms of RNA were used to synthesize cDNA using the SuperScript® III First-Strand Synthesis Systems (Invitrogen, Carlsbad, CA, USA). qRT-PCR was carried out with the ViiA™ 7 Real-Time PCR System (Applied Biosystems, Foster City, CA, USA). Sense and anti-sense primers targeted against the genes of interest are listed in Suppl Table 1. The transcript level of ribosomal protein L32 *(RPL32)* was used as an internal control.

### Small interfering RNA

Small interfering RNAs (siRNAs) targeting Tie-2 (J-003178-11 and J-003178-12) and Ang-1 (J-007802-07 and J-007802-08) as well as a scrambled RNA oligo were purchased from Dharmacon, Lafayette, CO, USA. Cells were transfected with the indicated siRNA using Lipofectamine RNAiMax (Invitrogen) following the manufacturer's instructions. Forty-eight hours after transfection, the cells were lysed for Western blotting analysis or for RNA extraction and qRT-PCR analysis.

### Flow cytometry analysis and fluorescence-activated cell sorting (FACS)

Cells were collected, washed twice with phosphate-buffered saline (PBS) and resuspended in 50 μl of FACS buffer (0.02% sodium azide and 2% FBS in PBS) before incubating with the fluorescent dye-conjugated antibodies at 4°C in the dark for 30 minutes. After incubation, the cells were washed twice with PBS and subsequently resuspended in 200 μl of FACS buffer. Flow cytometry analysis was performed using BD™ LSR II as described in the manufacturer's instruction manual, and the results were analyzed using KALUZA software.

For cell sorting, PC-3 cells were stained with Phycoerythrin (PE)-conjugated Tie-2 antibody in 200 μl of FACS buffer (2% FBS in PBS) at 4°C in the dark for 2 hours and the corresponding IgG isotype was used as negative control. After incubation, cells were washed twice with PBS and then resuspended in 500 μl of FACS buffer. The Tie-2^High^ population was sorted using the Beckman Coulter MoFloAstrios.

### Immunohistochemistry (IHC)

Sections rehydrated with standard procedures were incubated with 3% hydrogen peroxide (Sigma-Aldrich) for 10 minutes at room temperature. Antigen retrieval was performed with sodium citrate buffer at pH 6 in a pressure cooker for 10 minutes. Sections were then blocked with normal goat serum diluted in TBS for 1 hour. After the blocking, the sections were incubated with antibody against Tie-2 (1: 5000) (Santa Cruz Biotechnology, Dallas, TX, USA) for 1 hour, followed by a 1 hour incubation with biotinylated rabbit secondary antibody (Vector Laboratories, Burlingame, CA, USA) and a 30 minute incubation with VECTASTAIN® ABC Reagent complex. Signals were developed with the ImmPACT DAB Peroxidase (HRP) Substrate (Vector Laboratories). Slides were then counterstained with hematoxylin (Biocare Medical, Concord, CA, USA) before being mounted for analysis under the microscope.

### Generation of the stable Tie-2 overexpressing line

DU145 cells overexpressing Tie-2 (DU-Tie-2-GFP) were generated using the lentiviral gene delivery system as described in our previous study [44]. Briefly, pLenti6-Tie-2-GFP or the empty vector control was transfected into 293FT cells for lentiviral packaging. Viruses were collected and used to infect DU145 cells in the presence of polybrene (8μg/ml). Positive transfectants were selected with blasticidin (10μg/ml) and were further enriched by FACS based on the GFP signal.

### Western blot

Details regarding the experimental procedures have been described in our previous studies [45]. Briefly, whole cell lysates were prepared by lysing cell pellets with lysis buffer (Cell Signaling) containing 100 μM phenylmethylsulfonyl fluoride (PMSF; Sigma-Aldrich, St. Louis, MO, USA). The cell lysates were quantitated using the Pierce™ BCAProtein Assay *Kit* (Thermo Fisher Scientific, Rockford, IL, USA) before loading onto a SDS-polyacrylamide gel. The resolved proteins were then transferred onto a PVDF membrane (Millipore, Billerica, MA, USA), and the membrane was subsequently probed with the indicated antibody for 1 hour at room temperature prior to being washed with TBS-T buffer. The membrane was then incubated with the corresponding secondary antibodies for another hour at room temperature. After washing with TBS-T buffer, the membrane was incubated with Immobilon Western Chemiluminescent HRP Substrate (Millipore), and the signals were visualized using a Bio-Rad ChemiDoc™ XRS Gel Documentation System.

### Hoechst33342/Pyronin Y (HO/PY) quiescent staining

To determine the percentage of quiescent cells, all cells were first stained with 10 μg/ml HO for 45 minutes in the dark at 37°C. The cells were then incubated with 5 μM PY for another 45 minutes in the dark at 37°C as described in previous studies [46–48]. After staining, the cells were analyzed using BD™ LSR II, and the results were further analyzed using KALUZA software.

### Annexin V staining

To determine the percentage of apoptotic cells, Annexin V staining was performed using BD Pharmingen™ Annexin V-FITC kits following the manufacturer's instructions. Briefly, Tie-2^High^ and Tie-2^Low^ PC-3 cells were washed twice with cold PBS, resuspended in 100μl of 1X binding buffer and then incubated with Annexin V-FITC antibody and propidium iodide in the dark at room temperature for 15 minutes. After incubation, the apoptotic cells were analyzed using BD™ LSR II, and the results were further analyzed using KALUZA software.

### Ang-1 ELISA

To quantitate Ang-1 secretion by the prostate cancer cells, the cells were cultured in serum-free medium for 24 hours. The cells were harvested and counted using a Scepter^™^ Automated Cell Counter (Millipore). Conditioned medium was collected and concentrated using the 10K Amicon Ultra2-ml Centrifugal Filters (Millipore). The medium was analyzed using the Human Angiopoietin-1 DuoSet kits (R&D Systems, Minneapolis, MN, USA) following the manufacturer's instructions and the positive signals were determined using a LUMIstar OPTIMA Luminescence Microplate Reader and then, normalized it to the cell numbers.

### Cell adhesion assay

Prostate cancer cell lines were first labelled with HO for 45 minutes. Labelled cells (3 × 10^3^) were then overlaid directly onto confluent HUVECs, MG-63 cells or SaOS-2 cells. The cells were incubated for 30 minutes in the dark at 37°C. Unattached cells were dispersed with PBS, and the adherent cells were quantified using a LUMIstar OPTIMA Luminescence Microplate Reader. This experiment was repeated in the presence of 5 μM Tie-2 inhibitor or an equal volume of DMSO as a control. The data are presented as the fluorescence intensity from triplicate experiments.

### Animal studies

All animal studies were conducted in accordance with the Australian Code of Practice for the Care and Use of Animals for Scientific Purposes and were approved by the Animal Ethics Committee at the Queensland University of Technology (Approval No: 1100001393). Tumor metastasis were examined via intra-cardiac tumor cell injection using procedures described previously [23]. Briefly, the Tie-2^High^ population was isolated from PC3-Luc cells, which were stably transfected with a luciferase expression construct [44]. Three thousand Tie-2^High^ or Tie-2^Low^ cells were suspended in 100 μl of PBS before injecting into the left cardiac ventricle of 6-8-week-old male NOD-SCID mice (*n* = 8). Tumor metastasis were monitored every 2 weeks for a total of 8 weeks by intraperitoneal injection of D-luciferin (150 mg/kg) followed by bioluminescence imaging. The signal was detected using the Xenogen IVIS 100 imaging system. Mice were sacrificed at the end of the experiment, and *ex vivo* bioluminescence imaging was performed to confirm the incidence of metastasis. Statistical analysis was performed with the two-tailed *t* test, and differences were considered to be significant if *p* < 0.05.

## SUPPLEMENTARY MATERIALS AND METHODS


