# Supplementary material for: Tie-2 regulates the stemness and metastatic properties of prostate cancer cells
Source: Oncotarget. 2015 Apr 29;7(3):2572–84. doi: 10.18632/oncotarget.3950 (PMC4823056; doi:10.18632/oncotarget.3950)
Supplement: Supplementary file 1 [file oncotarget-07-2572-s001.pdf]

# Tie-2 regulates the stemness and metastatic properties of prostate cancer cells

## Supplementary Materials and Methods

### Cell lines and culture conditions

Prostate cancer cell lines PC-3, DU145, LNCaP and LAPC4 were obtained from ATCC (Rockville, MD, USA) and were maintained in RPMI 1640 medium (Invitrogen, Carlsbad, CA, USA) supplemented with 5% fetal bovine serum (FBS, Invitrogen) and 2% (wt/vol) penicillin-streptomycin P/S, Invitrogen); whereas 22Rv1 was a generous gift from Prof. Franky Chan (The Chinese University of Hong Kong) and was maintained in RPMI 1640 medium supplemented with 10% FBS and 2% (wt/vol) P/S. C42B was kindly provided by Prof Leland Chung (Cedars-Sinai Medical Center) and was maintained in T-Medium (Invitrogen) supplemented with 5% FBS and 2% P/S. MDA-PCa-2b was a kind gift from Dr Nora Navone (MD Anderson Cancer Center) and was maintained in BRFF-HPC1 medium (Athena Enzyme Systems) supplemented with 20% FBS and 1% P/S. Osteosarcoma cell lines MG-63 and SaOS-2 were obtained from ATCC and were maintained in Dulbecco's modified Eagle's medium (Invitrogen) (DMEM containing 10% FBS, 1% P/S) and McCoy's 5a medium (Invitrogen) containing 10% FBS, 1% P/S respectively. Human Umbilical Vein Endothelial Cells (HUVEC) were purchased from ScienCell Research Laboratories, Carlsbad, CA, USA and were maintained in endothelial culture medium (ECM) supplemented with 5% FBS, 1% endothelial cell growth factor (ECGS) and 1% P/S (ScienCell Research Laboratories). All cell types were kept at $37^0$C in a 5% CO2 environment.

### Antibodies and reagents

Tie-2 inhibitor, Hoechst33342 (HO) and Pyronin Y (PY) were purchased from Santa Cruz Biotechnology, Dallas, TX, USA. Recombinant Human Tie-2 Fc Chimera, CF was purchased from R&D Systems, Minneapolis, MN, USA and Human Ang-1 recombinant protein was purchased from PROSPEC, East Brunswick, NJ, USA. Gamma-tocotrienol (γ-T3) was provided by Davos Life Science Pty Ltd from Singapore, and was dissolved in absolute ethanol (100 mM). Cabazitaxel was purchased from Selleck, Houston, TX, USA and was dissolved in absolute ethanol (100 μM).

The following antibodies were used in this study: Phycoerythrin (PE) conjugated Tie-2 antibody and Mouse IgG1 PE Isotype Control (R&D Systems, Minneapolis, MN, USA); Human CD49f, pAKT and AKT antibodies (Cell Signalling Technology, Danvers, MA, USA); Bmi-1 antibody (Millipore, Billerica, MA, USA); p27 antibody (BD Biosciences); Tie-2, Actin and donkey anti-goat IgG-HRP antibody (Santa Cruz) and HRP conjugated anti-mouse and anti-rabbit secondary antibodies (GE Healthcare, Buckinghamshire, UK).

**Microarray analysis**

Duplicates of sorted Tie-$2^{Low}$ and Tie-$2^{High}$ populations were prepared for microarray profiling, which was performed on a custom Agilent 4X180k oligo array. The detailed experimental procedures have been described in a previous study [1].

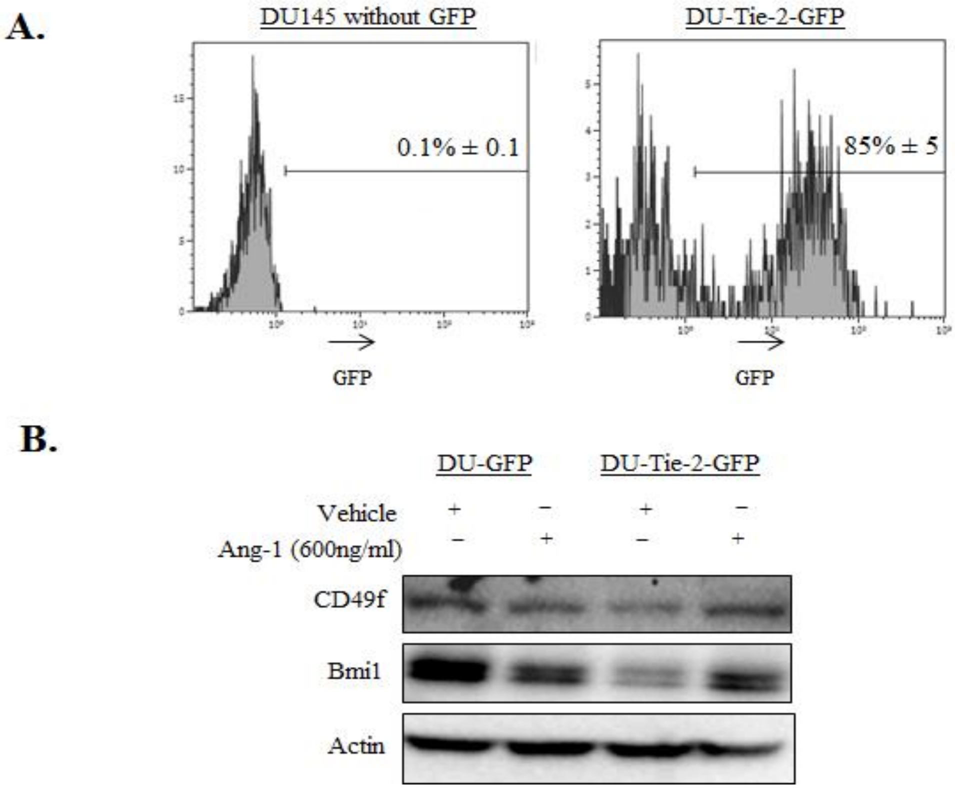


**Supplementary Figure 1: Ang-1 upregulated prostate CSC markers in DU-GFP-Tie-2 cells.** (A) DU145 constitutively expressing the Tie-2 protein (DU-Tie-2-GFP) was sorted by FACS using GFP as the marker. (B) Western blotting of prostate CSC markers (CD49f and Bmi-1) after Ang-1 treatment in DU-GFP and DU-Tie-2-GFP.

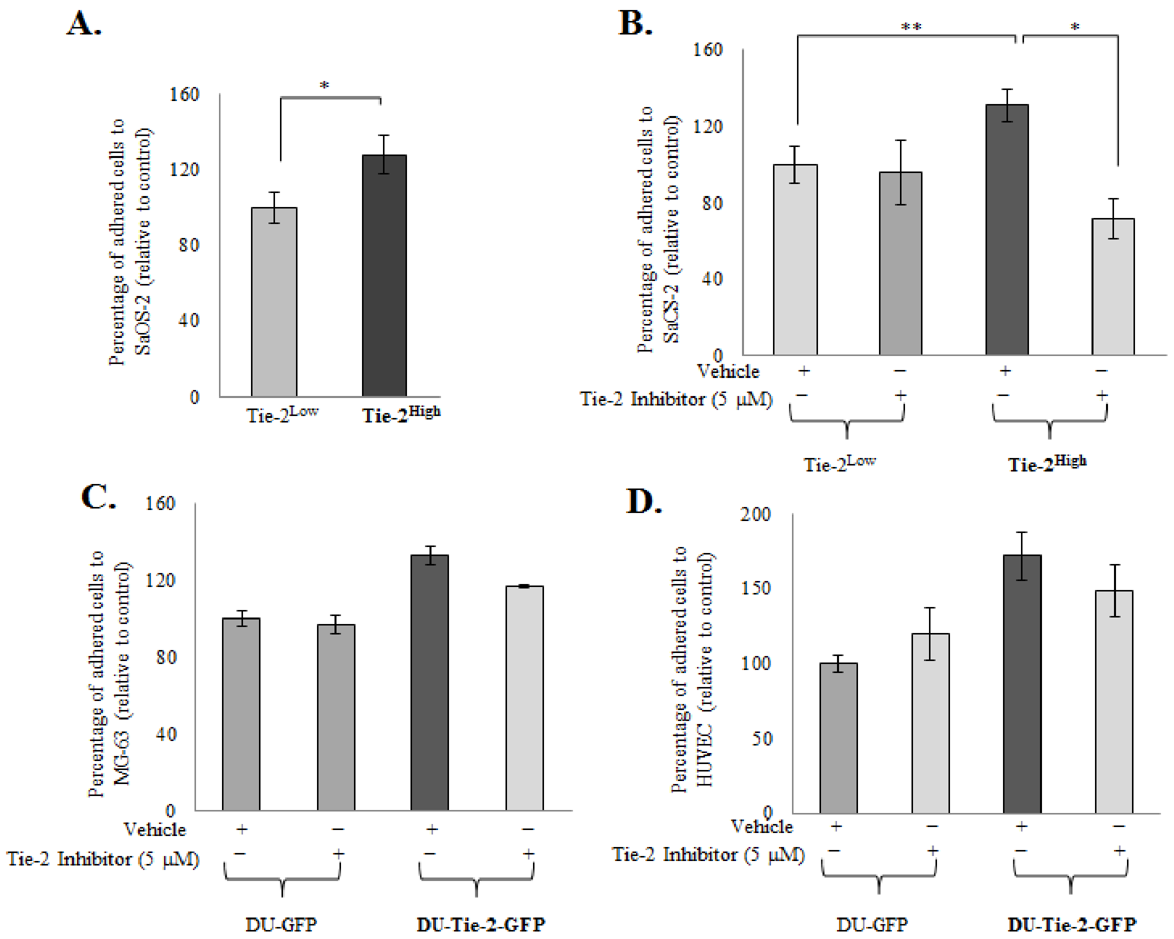


**Supplementary Figure 2: Tie-2 facilitated the adhesion of prostate cancer cells to bone (MG-63 and SaOS-2) and HUVEC cells.** (A) Tie-2$^{High}$ PC-3 cells were more adhesive to SaOS-2 cells when compared to Tie-2$^{Low}$ PC-3 cells. (B) Effect of Tie-2 inactivation on the adhesive ability of Tie-2$^{High}$ PC-3 cells. Treatment with a Tie-2 inhibitor (5 µM) prior to the adhesion assay significantly suppressed the adhesion ability of Tie-2$^{High}$ cells, while the same treatment failed to affect the Tie-2$^{Low}$ population. (C & D) The addition of Tie-2 inhibitor to DU-Tie-2-GFP cells drastically inhibited the adhesion ability of cells to MG-63 and HUVEC cells, but not in DU-GFP cells. Each experiment was repeated at least three times, and the results are presented as the mean ± SD.

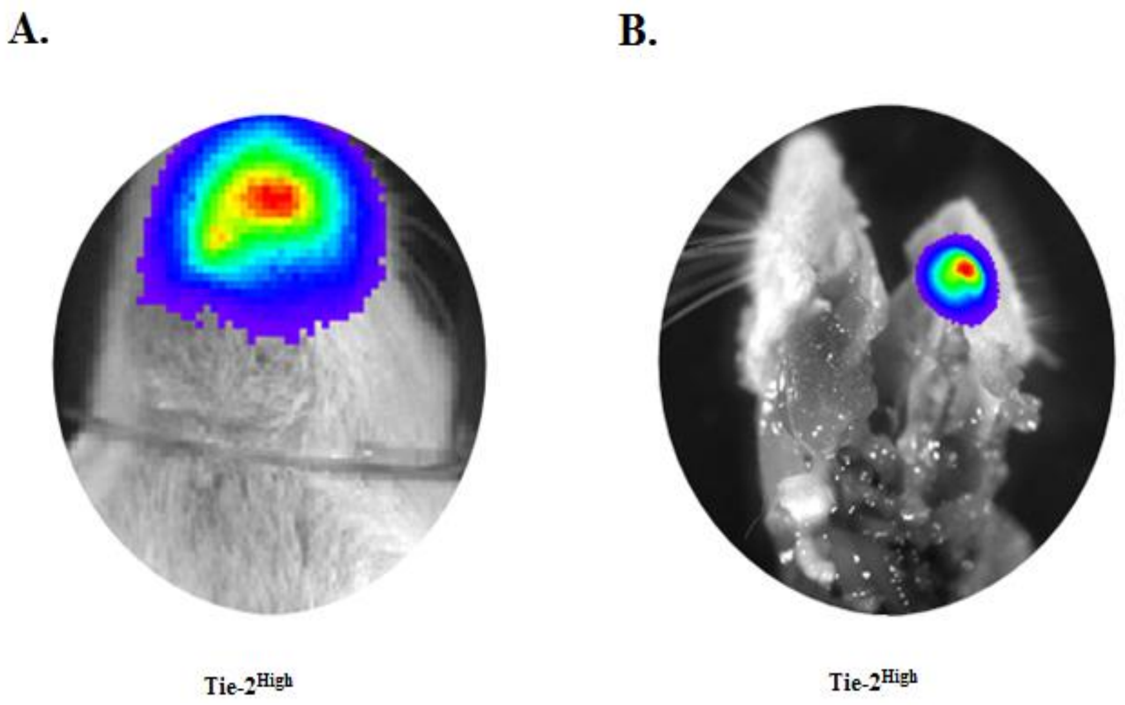


**Supplementary Figure 3: Localization of metastatic tumor in the mice with Tie-2$^{High}$ cell implantation.** (A) Bioluminescence images of mice with tumor metastasis at 8 weeks after the Tie-2$^{High}$ cell implantation. (B) Ex vivo imaging of the metastatic tumors. Note that, one of the mice exhibited jaw metastasis.

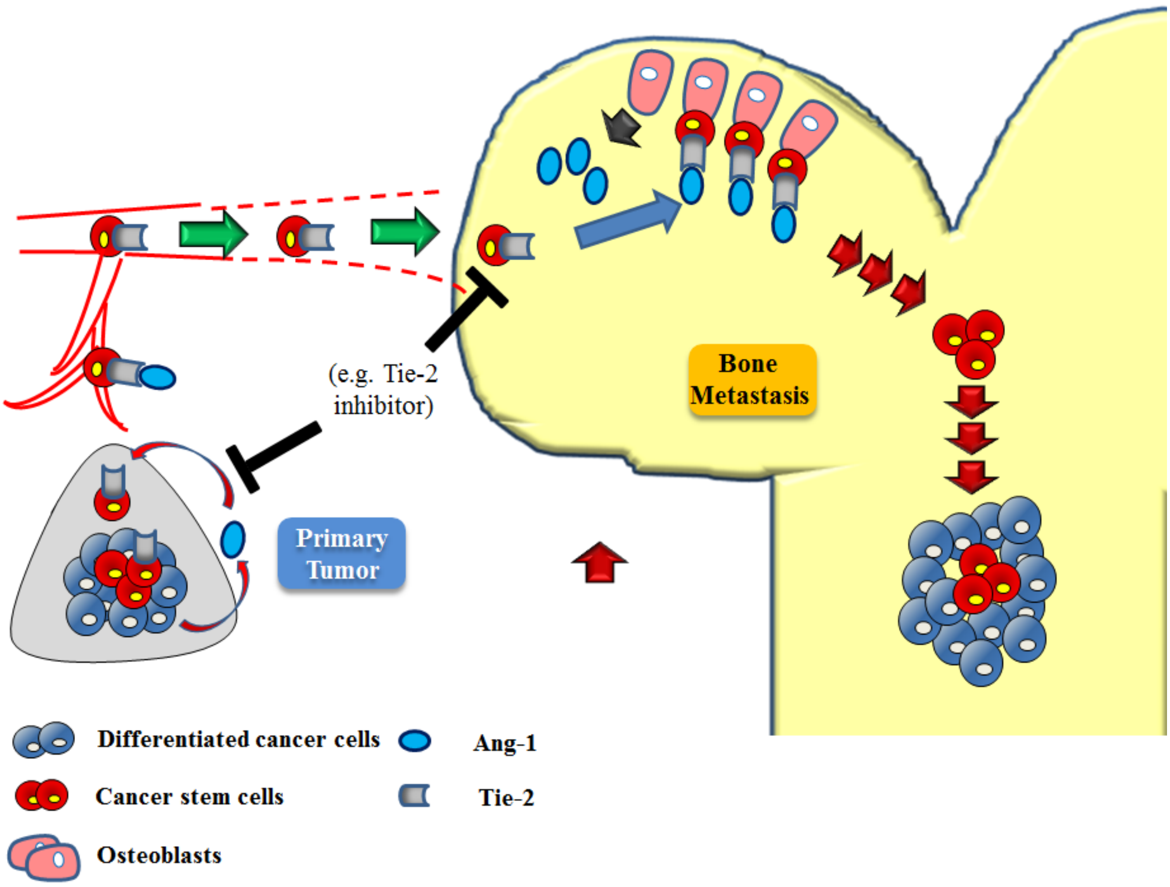


**Supplementary Figure 4: Model for the role of Tie-2 in prostate tumor metastasis.** Ang-1/Tie-2 functions as an autocrine loop that regulates the stemness and quiescence of a rare population of Tie-2 positive prostate cancer cells. These cells, which are capable of adhering to endothelial cells and obsteoblasts, are highly metastatic *in vivo* and may be responsible for mediating tumor metastasis *in vivo*. Thus, targeting the Ang-1/Tie-2 autocrine loop with Tie-2 inhibitor may offer opportunities for inhibiting prostate tumor metastasis by eliminating the metastatic cancer cell population.

**Supplementary Table 1: List of the primers used in this study.**

| Primer name | Sequence |
|---|---|
| Tie-2 forward | 5′-CTTTCTGGAACTGTGGAAGG-3′ |
| Tie-2 reverse | 5′-CTGGTGCTGGTTCATTAAGG-3′ |
| KITLG forward | 5′-CTGCTCCTATTTAATCCTCTCGT-3′ |
| KITLG reverse | 5′-TTGTACTACCATCTCGCTTATCC-3′ |
| CXCR4 forward | 5′- GCAGCAGGTAGCAAAGTGAC -3′ |
| CXCR4 reverse | 5′-AGAAGATGATGGAGTAGATGGTGG-3′ |
| FGF1 forward | 5′-ACAAGAAGCCCAAACTCCTC-3′ |
| FGF1 reverse | 5′-GTTCTCCTCCAGCCTTTCCA-3′ |
| ANGPT1 forward | 5′-ACGATGGCAACTGTCGTGAG-3′ |
| ANGPT1 reverse | 5′-TCCGACTTCATGTTTTCCACAA-3′ |

**Supplementary Table 2: Summary of cDNA microarray analysis.** The fold induction of CSC/HSC factors and markers in Tie-2$^{High}$ population when compared to Tie-2$^{Low}$ population as determined by cDNA microarray analysis. Each experiment was repeated at least twice.

| Gene Symbol | Description | RefseqID | Fold Change |
|---|---|---|---|
| **FGF1** | fibroblast growth factor 1 (acidic) (FGF1), transcript variant 6, mRNA. | NM_001144935 | **4.898439161** |
| CDKN1A | cyclin-dependent kinase inhibitor 1A (p21, Cip1) (CDKN1A), transcript variant 2, mRNA. | NM_078467 | **2.760138565** |
| PLAUR | plasminogen activator, urokinase receptor (PLAUR), transcript variant 2, mRNA. | NM_001005376 | **2.589319788** |
| PROM1 | prominin 1 (PROM1), transcript variant 4, mRNA. | NM_001145852 | **2.326933956** |
| **KITLG** | KIT ligand (KITLG), transcript variant a, mRNA. | NM_003994 | **2.311971311** |
| CD3D | CD3d molecule, delta (CD3-TCR complex) (CD3D), transcript variant 1, mRNA. | NM_000732 | **2.141386868** |
| FLOT2 | flotillin 2 (FLOT2), mRNA. | NM_004475 | **2.039587029** |
| CD38 | CD38 molecule (CD38), mRNA. | NM_001775 | **1.952222872** |
| TEK | TEK tyrosine kinase, endothelial (TEK), mRNA. | NM_000459 | **1.915497000** |
| BMP7 | bone morphogenetic protein 7 (BMP7), mRNA. | NM_001719 | **1.914510877** |
| **CXCR4** | chemokine (C-X-C motif) receptor 4 (CXCR4), transcript variant 1, mRNA. | NM_003467 | **1.902143786** |
| **ABCG2** | ATP-binding cassette, sub-family G (WHITE), member 2 (ABCG2), mRNA. | NM_004827 | **1.901389904** |
| FLT3 | fms-related tyrosine kinase 3 (FLT3), mRNA. | NM_004119 | **1.826128150** |
| CD24 | CD24 molecule (CD24), mRNA. | NM_013230 | **1.816880144** |
| IL8 | interleukin 8 (IL8), mRNA. | NM_000584 | **1.711807973** |
| SOX2 | SRY (sex determining region Y)-box 2 (SOX2), mRNA. | NM_003106 | **1.523596395** |

1. Sieh S, Taubenberger AV, Rizzi SC, Sadowski M, Lehman ML, Rockstroh A, An J, Clements JA, Nelson CC and Hutmacher DW. Phenotypic characterization of prostate cancer LNCaP cells cultured within a bioengineered microenvironment. PloS one. 2012; 7(9):e40217.
